# Dietary patterns and their associations with the occurrence and severity of early childhood caries among 1–5-year-olds in Iran: A multicenter cluster randomized survey

**DOI:** 10.1371/journal.pone.0319083

**Published:** 2025-02-25

**Authors:** Ehsan Javadzadeh, Samaneh Razeghi, Ahmadreza Shamshiri, Simin Z. Mohebbi

**Affiliations:** 1 Research Center for Caries Prevention, Dentistry Research Institute, Tehran University of Medical Sciences, Tehran, Iran; 2 Community Oral Health Department, School of Dentistry, Tehran University of Medical Sciences, Tehran, Iran; Shahid Beheshti University of Medical Sciences School of Dentistry, IRAN, ISLAMIC REPUBLIC OF

## Abstract

**Aim:**

A health-promoting and tooth-friendly diet can reduce the risk factors associated with early childhood caries (ECC) and promote overall health in children. This study aimed to identify dietary habits and feeding practices and their associations with ECC among 1–5-year-olds in Iran.

**Methods:**

This analytical cross-sectional study utilized data from a survey conducted from August 1, 2020, to January 20, 2021. A three-stage stratified cluster sampling method was employed to recruit participants. Parents provided written informed consent in Persian before enrollment and were then interviewed via a valid and reliable questionnaire inquiring about background information, feeding practices, and dietary habits. Each child underwent a dental examination by one of four trained and calibrated dentists to assess dental plaque and ECC. After appropriate weightings were applied, associations between key covariates and outcome measures were analyzed using multivariable logistic regression modeling and multivariate generalized negative binomial regression modeling. Statistical analysis was performed using SPSS V25 and Stata V14.2 software.

**Results:**

The mean age of the study population was 45.0 (±0.5) months, and 53% (±5%) had ECC. Visible dental plaque was present on the tooth surfaces of 39% ( ± 3%) of the toddlers and 66% ( ± 4%) of the preschoolers. The majority of the children had been breastfed for over 18 months (65%). Most children consumed sugar between meals once a day or less (66%) and fruits once (39%) or twice (37%) a day. ECC experience increased with age and poor oral hygiene across all age groups. Toddlers with a higher frequency of sugar intake between meals had higher dmft and ECC prevalence. The consumption of four to five servings of grain products per day was associated with a five-fold reduction in ECC occurrence (p = 0.047, OR = 0.2). In contrast, the consumption of two servings of fruits per day was associated with a doubling of the dmft score in toddlers (p = 0.015, IRR = 2.0).

**Conclusions:**

This study identified possible dietary risk factors for ECC in Iranian children, such as increased sugar intake and fruit consumption, while balancing diet with grain consumption played a protective role. Considering the role of oral hygiene and the multifactorial nature of the disease may highlight a route toward developing future targeted and effective preventive strategies.

## Introduction

Early childhood caries (ECC) are still considered a prominent health challenge globally among younger children, despite the general improvement in oral health practices. This is most likely related to their feeding practices and habits [1–3].

Owing to its epidemic prevalence in developing countries [3], ECC is a multifactorial disease involving the disposal of teeth and hosts, fermentable carbohydrates in the diet, cariogenic microorganisms, and time [4–6].

ECC remains a significant public health concern in Iran, affecting approximately 53.3% of children under six years of age [7]. Recent studies highlight the critical role of dietary patterns in the development and severity of ECC, underscoring the urgent need for targeted interventions and comprehensive research to address this pervasive issue [7,8].

Additionally, Iran’s Ministry of Health has reported a concerning increase in the consumption of sugary foods and beverages among young children, which directly contributes to rising ECC rates [9]. Addressing these dietary habits is crucial to curbing the growing epidemic of ECC.

Some key points from the World Health Organization (WHO) guidelines on dietary recommendations for the prevention of ECC include exclusive breastfeeding, reduced sugar intake, and a healthy diet [3]. Exclusive breastfeeding is for the first six months of life, followed by continued breastfeeding along with appropriate complementary foods up to two years of age or beyond [3]. Limiting the intake of free sugars to less than 10% of total energy intake is crucial. A further reduction to less than 5% of total energy intake provides additional health benefits [3].

A healthy diet could be provided by encouraging a diet rich in fruits, vegetables, and whole grains while avoiding sugary snacks and drinks [3]. The FDI World Dental Federation guidelines insist on a low cariogenic diet by promoting a tooth-friendly diet that minimizes the consumption of sugary foods and drinks [10]. These guidelines aim to reduce the risk factors associated with ECC and promote overall oral health in children.

A study by Liu et al. [11] investigated how the presence of cariogenic pathogens and different types of sugars impact dental biofilm composition and function. This highlights the roles of *Streptococcus mutans* and *Candida albicans* in the development of a dysbiotic/cariogenic oral microbiome, which is closely related to ECC. The American Academy of Pediatric Dentistry (AAPD) also noted that the interaction of cariogenic microorganisms and fermentable carbohydrates (such as sucrose) may induce demineralization, which can progress to cavitation. In children younger than three years, any sign of smooth-surface caries is indicative of severe ECC [4].

The relationship between breastfeeding and the risk of ECC has been a subject of research, and the findings suggest that breastfeeding itself is not an independent risk factor for ECC [12]. A study revealed that after adjusting for potential confounders, breastfeeding and breastfeeding duration were not associated with the risk for ECC [12]. Instead, factors such as poverty, being Mexican American, and maternal prenatal smoking were identified as independent risk factors for ECC [12]. Moreover, a meta-analysis [13] indicated that breastfed children are less affected by dental caries compared to bottle-fed children, suggesting that breastfeeding can protect against dental caries in early childhood. However, some studies have noted that breastfeeding beyond 12 months may increase the risk of caries, and reducing the frequency and number of nighttime feedings to mitigate this risk is recommended [14].

The relationship between the ECC and the consumption of sugar and fermentable carbohydrates is significant. Studies have shown that the intake of free sugars and fermentable carbohydrates increases the risk of ECC. A study revealed that children under two years of age who were given foods and beverages with free sugars had a higher prevalence of caries at age five [15]. The most recent guidelines published by the American Academy of Pediatrics (AAP) and Office of Disease Prevention and Health Promotion (ODPHP) ban the use of added sugar for children under two years of age [16,17].

The intake of fruit juice with meals and cookies or biscuits at 24 months of age, as well as fast food consumption at least once every week, more than doubled the risk for ECC [15]. Snacks with fermentable carbohydrates can alter oral pH levels, which may affect the colonization of cariogenic microorganisms such as *Streptococcus mutans* and *Candida albicans*. This can lead to demineralization and, over time, the loss of tooth structure or cavitation [18,19]. Frequent consumption of such snacks and beverages increases the risk of caries due to prolonged contact between sugars and cariogenic bacteria on susceptible teeth [20].

Several studies have explored the associations between dietary patterns and ECC, but the findings remain inconsistent [21]. For example, a study by van Meijeren-van Lunteren et al. (2023) revealed that adherence to dietary guidelines was associated with a lower occurrence of severe dental caries among children [22]. However, this association was weakened when adjusted for oral hygiene practices, suggesting that dietary patterns alone may not be sufficient to prevent ECC without proper oral care [22].

Another study by Hu et al. (2018) examined dietary patterns in a multiethnic Asian cohort and reported that following WHO weaning guidelines was protective against ECC development [21]. This contrasts with other studies emphasize the role of frequent sugar consumption in ECC progression [8]. These conflicting results highlight the need for further research to clarify the complex interplay between dietary habits, oral hygiene, and ECC.

There are notable gaps in the literature regarding the long-term effects of early dietary interventions on ECC prevention. Most studies focus on short-term outcomes, leaving a critical gap in understanding how sustained dietary changes impact ECC over time. Addressing these gaps is crucial for developing effective ECC prevention strategies.

Furthermore, the influence of socioeconomic status on dietary habits and ECC occurrence remains underexplored, particularly in low- and middle-income countries such as Iran. This represents a critical area for future research to ensure equitable health outcomes.

With respect to some controversial findings about the role of diet in oral and general health during early childhood [23–25], the present study aims to comprehensively investigate dietary patterns and their associations with ECC among Iranian children aged 1–5 years old.

## Methods

### Study design and population

The present descriptive, analytical cross-sectional study provided data from a survey conducted from August 1, 2020, to January 20, 2021, that targeted Iranian children aged 1–5 years and their parents and used a detailed methodological approach to assess dietary habits and feeding practices at different socioeconomic levels, urban and rural areas, and associations with ECC [26].

### Sampling method

The study used a three-stage stratified cluster sampling method to recruit participants. This method likely involves dividing the population into strata according to the severity of ECC reported in previous national data [27], then randomly selecting clusters within those strata, and finally randomly selecting individuals within those clusters. Four randomly representative provinces were selected: Razavi Khorasan, West Azerbaijan, Yazd, and Qom.

Leveraging the authority delegated to executive implementers and administrative coordinators, a comprehensive list of public centers and reference sites was compiled to maximize the sampling of children within the study’s target age range. This list included preschools, kindergartens, elementary schools, health centers, mother and child health care and vaccination units, health houses (Iran’s primary health care units for rural populations), community centers, and other public gathering sites, especially in less privileged areas. The study’s final 55 clusters consisted of 37 urban and 18 rural sites from the compiled list. Families in each randomly selected city or village were invited through these centers to learn about the study’s objectives, benefits, and participation process. Each center coordinated a date (usually 15 days after the initial contact) for interviews with interested parents and dental examinations for their children.

Efforts were made to ensure that samples were collected from multiple centers or sites in each city or village to minimize potential selection bias.

To mitigate the effects of over- or undersampling of specific population groups (sampling bias), study data were weighted to adjust for respondents’ relative contributions based on known population characteristics, including age, gender, and location of residence, according to Iran’s 2016 population census [28].

The interview-based method of obtaining responses to the questionnaire items greatly reduced the likelihood of nonresponse bias and, to some extent, recall bias.

In coordination with each center, especially kindergartens and preschools, efforts were made to obtain parental consent for all children within the target age range to eliminate potential volunteer bias.

Regarding possible attrition bias, participant dropout was minimal and primarily attributable to restrictions during the COVID-19 pandemic [26].

The participating children were divided into five age groups: 12–23, 24–35, 36–47, 48–59, and 60–71 months of age. For additional analyses, the children were recategorized into two groups: 12–47 months (toddlers) and 48–71 months (preschoolers).

### Data collection

#### Parent interviews.

Participation in the study was voluntary, and each participant had the option to withdraw from the study at any stage of the study. Parents or guardians provided signed written informed consent in Persian before enrollment and were then interviewed via the study questionnaire.

To minimize the possibility of any bias during data collection, questionnaire responses were obtained through face-to-face interviews. During the interview, each question was asked orally, and clarifications were provided if parents had difficulty in understanding or had questions about any items. The responses were recorded directly into the questionnaire by the researcher or an assistant [26].

The questionnaire was primarily based on a previously used English-language instrument [29,30], which was revised during translation into Persian to ensure its intended meaning and align with the study objectives. The content validity of the questionnaire was agreed upon by a panel of experts in community oral health and pediatric dentistry after some modifications were made to some controversial items. The content validity index (CVI) and content validity ratio (CVR) exceeded 0.75 for all items. To ensure the content accuracy, similarity, and clarity of the Persian version compared with the original English questionnaire, two independent dental faculty experts confirmed its face validity. Additionally, pilot testing was conducted to evaluate the test-retest reliability and to assess the face validity of the questionnaire from the perspective of the interviewees. This pilot test was conducted on separate days with a sample of 20 parents who were excluded from the final study sample. The actual agreement for all the questions exceeded 90%. No changes to the questions or responses were necessary following the pilot study [26].

The questionnaire included the following parts:

I Background information: Demographics and socioeconomic characteristics, including gender, age, and location of residence (urban/rural)
II Feeding practices: breastfeeding duration, bottle feeding duration
III Dietary habits: frequency of sugar intake between meals, vegetables, fruits, foods rich in protein, grain products, dairy products, and fast foods among Iranian toddlers and preschoolers.

During interviews with parents to obtain responses to the questionnaire items, each group of edible materials and corresponding foodstuffs was thoroughly explained and defined.

Furthermore, parents were informed that any processed or unprocessed, natural or synthetic foodstuff or drink with a sweet taste, or even those not necessarily sweet but containing sugar ingredients, was considered sugar-containing. Sugars can be either naturally occurring or added. Natural sugars are found in fruits and vegetables, while added sugars, such as table sugar and high-fructose corn syrup, are incorporated into foods and drinks during processing and preparation to enhance flavor. Additionally, natural sweeteners such as honey and maple syrup, which contain glucose and fructose, are also used to sweeten foods. Processed foods, including sweets, beverages, and condiments such as ketchup and dressings, often contain added sugars.

Protein-rich foods were defined as meat (e.g., lamb, chicken, fish), nuts (e.g., almond), eggs, soy products, lentils, beans, peas, and similar items. Dairy products, including yogurt and cheese, were listed as separate items from protein-rich foods in the questionnaire.

#### Dental examinations.

Each researcher was responsible for conducting the child’s dental examination after completing the questionnaire through a face-to-face interview with the parents.

Oral examinations were conducted via the protocol for ECC diagnosis and risk assessment proposed by Evans et al. in 2018 [31]. The ECC was defined according to the AAPD definition [4,32].

Each child received a dental examination conducted by one of four trained and calibrated dentists, each assisted by a data recorder. These examinations assessed the presence of ECC and its severity using the decayed, missing, and filled teeth index (dmft score) [26].

Prior to data collection, the second author, a specialist in pediatric dentistry, was established as the gold standard during the calibration process. Calibration sessions were then conducted between the examiners, the first author, and the specialist using a sample of 50 preschool children aged 36–71 months (excluded from the final study sample) as part of a pilot study.

To assess intra-examiner reliability, dental examinations were repeated one hour later. Calibration training focused on precise plaque level assessment on a surface-by-surface basis, diagnosing ECC, and distinguishing ECC from other lesions, such as developmental defects, abnormalities, and noncarious opacities (e.g., deciduous molar hypomineralization, demarcated opacities, and enamel hypoplasia) [26].

The calibration process involved calculating kappa coefficients for ECC to determine inter- and intra-examiner agreement. During the pilot study and subsequent recalibration sessions (held every six months throughout the study), kappa coefficients were consistently maintained at κ ≥ 0.85. This high level of inter- and intra-examiner reliability (κ ≥ 0.85) ensured consistency in the examinations [26].

The highest plaque level observed on a child’s dental surface was recorded using the Silness and Löe plaque index [33,34], coded from 0 to 3, corresponding to the surface with the highest score. For ease of analysis, plaque scores were recoded and dichotomized as visible or nonvisible [35]. This simplified coding approach was used not only for descriptive statistics but also for regression analysis [26]. Since the number of teeth in young children varies with age, the presence of plaque on any primary tooth surface was used as a proxy for the child’s oral hygiene status.

Since the majority of the target children were found in daycare settings such as preschools, kindergartens, health centers, mother and child health care and vaccination units, and health houses, most oral and dental examinations were conducted during early working hours [26].

### Statistical analysis

The questionnaire and oral examination data were entered into Microsoft Excel V2207 and then converted to SPSS V25 and Stata V14.2 formats. All identifiable personal information was adequately disguised in the data to preserve the individuals’ anonymity. The descriptive data are reported as frequencies and percentages for qualitative variables and means with standard errors for quantitative variables.

The associations between key covariates and primary outcomes were assessed using multivariate logistic regression modeling and multivariate generalized negative binomial regression modeling. These statistical methods are suitable for examining the relationships between multiple independent variables (dietary habits and feeding practices associated variables) and a dependent variable (ECC occurrence and the dmft score as an indicator for ECC severity).

As mentioned before, the analysis took into account the complex survey design by applying appropriate weightings. Weights were calculated and applied to certain demographic variables, including age, gender, and location, so they could be compared with known characteristics of the general population of almost seven million Iranian children aged 12 to 71 months obtained from Iran’s latest population census report in 2016 [28]. A p-value of < 0.05 was considered statistically significant.

### Ethics approval and considerations

The project was approved by the Ethics Committee of Tehran University of Medical Sciences in accordance with ethical principles, national norms, and standards for conducting medical research in Iran (Approval ID: IR.TUMS.DENTISTRY.REC.1398.070).

Participation in the study was voluntary, and participants had the right to withdraw at any stage. Written informed consent was obtained from the parents in Persian before enrollment. To ensure confidentiality, all identifiable personal information was adequately anonymized in the data to preserve the individuals’ privacy.

## Results

### Background information

A total of 909 children were included in the study, 96% of whom underwent a clinical examination. This sample consisted of 468 toddlers and 441 preschoolers. Additionally, 346 children were from rural areas, and 563 were from urban areas. Among all the children, 467 were girls, and 442 were boys. The mean ages of the toddlers, preschoolers, and the entire study population were 31.5 (±0.5), 59.2 (±0.3), and 45.0 (±0.5) months, respectively. Table 1 shows the distribution of the studied children according to gender, age group, and location of residence.

**Table 1:** Distribution of Iranian 1- to 5-year-olds (n = 909) according to gender, age group, and location of residence.

Location of Residence			Urban			Rural			Total		
Age Group		Gender	Count	Percent*	SE* (%)	Count	Percent*	SE* (%)	Count	Percent*	SE* (%)
**Toddler**	**12 − 23 months**	**Female**	34	4	1	29	3	1	63	7	1
		**Male**	43	5	1	27	3	1	70	8	1
		**Total**	77	8	1	56	6	1	133	15	1
	**24 − 35 months**	**Female**	44	5	1	40	4	1	84	9	1
		**Male**	37	4	1	27	3	1	64	7	1
		**Total**	81	9	1	67	7	1	148	16	1
	**36 − 47 months**	**Female**	66	7	1	39	4	1	105	12	1
		**Male**	55	6	1	27	3	1	82	9	1
		**Total**	121	13	1	66	7	1	187	21	1
	**Total** **(12 − 47 months)**	**Female**	144	16	1	108	12	1	252	28	1
		**Male**	135	15	1	81	9	1	216	24	1
		**Total**	279	31	2	189	21	1	468	51	2
**Preschooler**	**48 − 59 months**	**Female**	69	8	1	40	4	1	109	12	1
		**Male**	69	8	1	49	5	1	118	13	1
		**Total**	138	15	1	89	10	1	227	25	1
	**60 − 71 months**	**Female**	70	8	1	36	4	1	106	12	1
		**Male**	76	8	1	32	4	1	108	12	1
		**Total**	146	16	1	68	7	1	214	24	1
	**Total** **(48 − 71 months)**	**Female**	139	15	1	76	8	1	215	24	1
		**Male**	145	16	1	81	9	1	226	25	1
		**Total**	284	31	2	157	17	1	441	49	2
**All Ages**	**(12 − 71 months)**	**Female**	283	31	2	184	20	1	467	51	2
		**Male**	280	31	2	162	18	1	442	49	2
		**Total**	563	62	2	346	38	2	909	100	

SE: Standard error.

*No weighting was applied to adjust the relative contribution of the respondents using known populational characteristics.

Overall, 53% (±5%) of the children had ECC, 35% (±5%) of toddlers, and 83% (±3%) of preschoolers. The ECC prevalence was 44% ( ± 5%) in the urban population and 61% ( ± 4%) in the rural population. The overall mean dmft score for the whole sample of children was 2.7 ( ± 0.3). The mean dmft score was 1.1 ( ± 0.2) for toddlers and 5.3 ( ± 0.3) for preschoolers.

Overall, 49% ( ± 4%) of the children had visibly detectable dental plaque. Plaques were visible on dental surfaces of 39% ( ± 3%) of the toddlers compared with 66% ( ± 4%) of the preschoolers. Although plaque prevalence generally increased with age, both preschooler subgroups (48–59 and 60–71 months) had the same prevalence of visible plaque, at 66% ( ± 5%).

Children with visible dental plaque, across all age groups, had significantly higher ECC prevalence and mean dmft scores.

Table 2 shows the prevalence of visually detectable dental plaque according to age group and location of residence.

**Table 2:** Prevalence of visually detectable dental plaque among Iranian 1- to 5-year-olds (n = 909) according to age group and location of residence.

Location of Residence		Urban					Rural					Total				
Visually Detectable Dental Plaque		Prevalence* (%)	SE* (%)	95% CI*		Cases	Prevalence* (%)	SE* (%)	95% CI*		Cases	Prevalence* (%)	SE* (%)	95% CI*		Cases
				Lower	Upper				Lower	Upper				Lower	Upper	
**Toddler**	12** − **23 months	19	6	8	31	15	14	3	7	21	11	18	4	9	26	26
	24** − **35 months	47	4	39	56	40	50	8	33	67	33	48	4	41	56	73
	36** − **47 months	39	7	25	54	48	79	7	64	93	48	51	5	40	62	96
	Total (12** − **47 months)	35	4	28	43	103	47	5	38	56	92	39	3	33	45	195
**Preschooler**	48** − **59 months	61	7	47	74	80	78	5	68	88	70	66	5	56	75	150
	60** − **71 months	57	7	43	70	81	89	5	79	99	59	66	5	56	77	140
	Total (48** − **71 months)	59	6	46	71	161	83	3	77	90	129	66	4	57	75	290
**All Ages**	(12** − **71 months)	44	5	35	54	264	61	4	54	68	221	49	4	42	56	485

SE: Standard error, CI: confidence interval.

*Weighting was used to adjust the relative contribution of the respondents using known populational characteristics, including age, gender, and location of residence, according to Iran’s latest population census in 2016.

### Feeding practices and dietary habits

Most children had been breastfed for more than 18 months (65%), 29% had been breastfed for 1 to 18 months, and 6% had never been breastfed. The duration of breastfeeding was almost similar among rural and urban children. Compared with rural children, children in urban areas experienced a greater prevalence and longer duration of bottle feeding. Most children consumed sugar between meals once a day or less (66%). There was no significant difference in this consumption pattern between either rural and urban children or between toddlers and preschoolers. Table 3 provides detailed information on the duration of breastfeeding, bottle feeding, sugar intake, and other dietary habits across different age groups.

**Table 3:** Distribution of Iranian 1- to 5-year-olds (n = 909) according to prevalence * (%) of dietary habits and feeding practices.

		Toddler (12 − 47 months)				Preschooler (48 − 71 months)			All Ages (12 − 71 months)
		12 − 23 months	24 − 35 months	36 − 47 months	Total	48 − 59 months	60 − 71 months	Total	
**Breastfeeding Duration**	**Never**	7	8	4	6	8	1	5	6
	**1 − 18 month(s)**	52	29	24	35	20	20	20	29
	**>18 months**	42	63	72	59	71	79	75	65
**Bottle Feeding Duration**	**Never**	68	59	72	72	54	54	65	66
	**1 − 12 month(s)**	23	14	6	6	18	18	14	14
	**>12 months**	9	27	21	21	29	29	21	20
**Sugar Intake Between Meals**	**Never**	35	14	8	20	9	11	10	16
	**Once a Day or Less**	59	59	78	65	70	66	68	66
	**Twice a Day**	5	20	11	12	15	18	17	14
	**Three Times a Day or More**	1	6	3	3	5	5	5	4
**Dairy Products**	**Once a Day or Less**	47	38	52	46	35	41	38	43
	**Twice a Day**	46	39	34	40	48	41	45	42
	**Three Times a Day**	4	17	11	11	13	14	13	12
	**More than Three Times a Day**	3	5	2	3	4	3	4	3
**Foods Rich in Protein**	**Never**	17	7	1	9	2	3	3	6
	**Several Times a Week**	46	49	53	49	55	54	54	51
	**Once a Day**	16	18	16	17	23	21	22	19
	**Twice a Day**	19	23	26	22	17	18	18	21
	**More than Twice a Day**	2	4	3	3	3	3	3	3
**Grain Products**	**Less than Twice a Day**	58	21	35	38	25	38	31	36
	**Two to Three Times a Day**	38	70	57	55	67	53	60	57
	**Four to Five Times a Day**	2	9	9	6	8	8	8	7
	**More than Five Times a Day**	2	0	0	1	1	0	1	1
**Fruits**	**Never**	25	10	8	15	1	3	2	10
	**Once a Day**	34	41	48	41	42	33	37	39
	**Twice a Day**	36	34	29	33	41	43	42	37
	**Three Times a Day or More**	6	15	14	12	16	21	18	15
**Vegetables**	**Never**	52	27	23	35	18	25	21	30
	**Once a Day**	39	56	62	52	63	60	62	56
	**Twice a Day**	6	14	10	10	16	10	13	11
	**More than Twice a Day**	2	2	5	3	3	5	4	3
**Fast Foods**	**Once a Month or Less**	87	71	72	77	63	69	66	72
	**Several Times a Month**	12	29	28	23	35	30	32	27
	**Two to Three Times a Week**	1	0	1	0	3	1	2	1

*Weighting was used to adjust the relative contribution of the respondents using known populational characteristics, including age, gender, and location of residence, according to Iran’s latest population census in 2016.

The majority of Iranian children consumed dairy products twice a day or less (85%), with no clear difference between toddlers and preschoolers. However, the frequency of dairy consumption increased negligibly with age. Across all age groups, the most common frequency for consuming foods rich in protein was “Several Times a Week” (51%), whereas the least common frequency was “More than Twice a Day” (3%).

For grain products, “Two to Three Times a Day” was the most frequent feeding pattern across all age groups except for children aged 12–23 months, with “More than Five Times a Day” being the least common. Moreover, “Once a Day” and “Twice a Day” were the most prevalent frequencies of fruit consumption across all age groups. The most common frequency for vegetable consumption was “Once a Day”, whereas the least common frequency was “More than Twice a Day”. Among all the age groups studied, the most common frequency for consuming fast foods was “Once a Month or Less”, whereas the least common frequency was “Two to Three Times a Week”. Additionally, the frequency of fast food consumption increased with age.

### ECC prevalence and severity and association with dietary patterns

A significant increase in ECC prevalence and a less notable increase in the mean dmft score were observed only in toddlers with a longer duration of breastfeeding. Additionally, the ECC prevalence increased significantly with increasing frequency of sugar intake between meals, which was observed only in toddlers (from 19% to 68%) (Table 4).

**Table 4:** Early childhood caries (ECC) prevalence and mean dmft score among Iranian 1- to 5-year-olds (n = 909) according to breastfeeding duration, bottle feeding duration, and sugar intake frequency between meals.

			Early Childhood Caries (ECC) Prevalence					Mean dmft Score				
	Age Group	Frequency	Prevalence* (%)	SE* (%)	95% CI*		Cases	Mean*	SE*	95% CI*		Count
					Lower	Upper				Lower	Upper	
**Breastfeeding Duration**	Toddler (12** − **47 months)	Never	18	6	5	31	7	0.6	0.3	0.1	1.2	28
		1** − **18 month(s)	28	5	18	38	47	0.8	0.2	0.4	1.2	166
		>18 months	41	7	27	55	125	1.4	0.2	0.9	1.9	274
	Preschooler (48** − **71 months)	Never	72	10	51	93	20	4.1	0.8	2.6	5.7	26
		1** − **18 month(s)	72	9	55	89	69	4.5	0.7	3.2	5.9	94
		>18 months	86	2	82	91	272	5.6	0.3	5.0	6.1	321
	All Ages (12** − **71 months)	Never	35	7	22	49	27	1.8	0.3	1.1	2.4	54
		1** − **18 month(s)	39	6	28	51	116	1.8	0.3	1.1	2.4	260
		>18 months	61	6	50	73	397	3.2	0.3	2.5	3.9	595
**Bottle Feeding Duration**	Toddler (12** − **47 months)	Never	36	6	23	48	119	1.1	0.2	0.7	1.4	314
		1** − **12 month(s)	25	6	12	38	23	0.8	0.2	0.3	1.3	68
		>12 months	40	9	22	57	37	1.6	0.4	0.7	2.4	86
	Preschooler (48** − **71 months)	Never	85	3	80	91	246	5.5	0.3	4.9	6.1	294
		1** − **12 month(s)	71	10	51	90	45	3.7	0.7	2.3	5.1	60
		>12 months	83	5	72	94	70	5.6	0.6	4.4	6.7	87
	All Ages (12** − **71 months)	Never	54	6	43	66	365	2.7	0.3	2.1	3.4	608
		1** − **12 month(s)	42	7	28	55	68	1.9	0.4	1.1	2.7	128
		>12 months	58	8	42	73	107	3.2	0.5	2.2	4.3	173
**Sugar Intake Frequency Between Meals**	Toddler (12** − **47 months)	Never	19	5	8	30	21	0.6	0.2	0.2	0.9	85
		Once a Day or Less	33	5	22	44	111	1.0	0.2	0.6	1.4	308
		Twice a Day	62	8	46	78	34	2.4	0.5	1.4	3.4	58
		Three Times a Day or More	68	16	35	101	13	2.0	0.5	0.9	3.0	17
	Preschooler (48** − **71 months)	Never	85	6	73	97	45	6.2	1.0	4.2	8.1	51
		Once a Day or Less	82	4	74	91	234	5.0	0.4	4.3	5.8	292
		Twice a Day	82	6	70	94	62	5.4	0.9	3.6	7.2	75
		Three Times a Day or More	85	11	63	108	19	5.7	1.0	3.8	7.7	22
	All Ages (12** − **71 months)	Never	35	6	24	46	66	1.9	0.4	1.2	2.6	136
		Once a Day or Less	52	6	41	64	345	2.6	0.3	2.0	3.2	600
		Twice a Day	71	7	58	85	96	3.8	0.7	2.4	5.1	133
		Three Times a Day or More	77	10	56	97	32	3.8	0.8	2.2	5.3	39

SE: Standard error, CI: confidence interval.

*Weighting was used to adjust the relative contribution of the respondents using known populational characteristics including age, gender, and location of residence according to Iran’s latest population census in 2016.

In terms of the prevalence of ECC and the mean dmft score, no significant difference was observed with respect to the frequency of consumption of dairy products, protein-rich foods, or grain products. However, among toddlers, a higher prevalence of ECC and a higher mean dmft score were observed in those consuming one to two servings of fruits per day (Table 5).

Among toddlers, the ECC prevalence was nearly twice as high in those who consumed vegetables “More than Twice a Day” (64%) than in those who “Never” consumed vegetables (31%). However, this was not reflected in the mean dmft scores (1.0 vs. 0.9).

The highest mean dmft scores were found among both toddlers and preschoolers who consumed fast foods “Several Times a Month”, with scores of 1.3 (±0.2) for toddlers and 5.4 (±0.4) for preschoolers (Table 5).

**Table 5:** Early childhood caries (ECC) prevalence and mean dmft score among Iranian 1- to 5-year-olds (n = 909) according to the consumption frequency of dairy products, foods rich in protein, grain products, fruits, vegetables, and fast foods.

			Early Childhood Caries (ECC) Prevalence					Mean dmft Score				
	Age Group	Frequency of Feeding	Prevalence* (%)	SE* (%)	95% CI*		Cases	Mean*	SE*	95% CI*		Count
					Lower	Upper				Lower	Upper	
**Dairy Products**	**Toddler** **(12 − 47 months)**	**Once a Day or Less**	36	6	23	48	84	1.1	0.2	0.8	1.4	223
		**Twice a Day**	35	6	23	47	70	1.2	0.3	0.7	1.7	179
		**Three Times a Day**	37	9	19	54	21	1.2	0.3	0.5	1.8	51
		**More than Three Times a Day**	21	14	**−**7	50	4	1.1	0.6	**−**0.2	2.4	15
	**Preschooler** **(48 − 71 months)**	**Once a Day or Less**	83	5	73	92	136	5.3	0.6	4.1	6.6	169
		**Twice a Day**	83	4	75	91	164	5.3	0.4	4.6	6.1	198
		**Three Times a Day**	87	6	76	99	48	5.3	0.9	3.5	7.1	56
		**More than Three Times a Day**	66	15	37	96	13	3.8	1.1	1.5	6.1	18
	**All Ages** **(12 − 71 months)**	**Once a Day or Less**	52	6	39	64	220	2.5	0.4	1.8	3.3	392
		**Twice a Day**	55	6	42	67	234	2.9	0.4	2.1	3.7	377
		**Three Times a Day**	59	8	42	75	69	3.0	0.6	1.8	4.1	107
		**More than Three Times a Day**	39	13	12	65	17	2.1	0.8	0.6	3.7	33
**Foods Rich in Protein**	**Toddler** **(12 − 47 months)**	**Never**	15	6	4	27	9	0.3	0.1	0.1	0.6	44
		**Several Times a Week**	43	6	30	55	104	1.5	0.2	1.0	2.0	240
		**Once a Day**	28	5	18	39	35	1.0	0.2	0.5	1.5	96
		**Twice a Day**	29	7	14	43	24	0.7	0.2	0.4	1.0	74
		**More than Twice a Day**	51	17	16	86	7	0.7	0.2	0.3	1.2	14
	**Preschooler** **(48 − 71 months)**	**Never**	100	0	100	100	16	5.6	0.5	4.5	6.6	16
		**Several Times a Week**	83	4	75	92	194	5.6	0.5	4.7	6.5	231
		**Once a Day**	77	6	64	89	84	5.1	0.6	4.0	6.2	111
		**Twice a Day**	85	4	77	92	51	4.9	0.4	4.1	5.7	65
		**More than Twice a Day**	93	5	83	103	16	3.3	0.7	1.9	4.6	18
	**All Ages** **(12 − 71 months)**	**Never**	28	7	13	43	25	1.1	0.4	0.4	1.9	60
		**Several Times a Week**	59	6	47	71	298	3.2	0.4	2.4	3.9	471
		**Once a Day**	50	5	40	60	119	2.9	0.4	2.0	3.7	207
		**Twice a Day**	47	8	32	62	75	2.1	0.3	1.4	2.8	139
		**More than Twice a Day**	67	14	39	95	23	1.7	0.3	1.0	2.4	32
**Grain Products**	**Toddler** **(12 − 47 months)**	**Less than Twice a Day**	33	7	19	47	66	0.9	0.2	0.5	1.4	176
		**Two to Three Times a Day**	38	5	28	48	103	1.3	0.2	0.9	1.8	254
		**Four to Five Times a Day**	23	8	7	40	10	0.7	0.2	0.3	1.2	33
		**More than Five Times a Day**	0	0	0	0	0	0.0	0.0	0.0	0.0	5
	**Preschooler** **(48 − 71 months)**	**Less than Twice a Day**	84	5	74	95	108	6.1	0.6	5.0	7.3	131
		**Two to Three Times a Day**	82	3	76	88	215	4.9	0.3	4.4	5.5	264
		**Four to Five Times a Day**	83	8	67	99	33	4.7	0.7	3.3	6.0	40
		**More than Five Times a Day**	87	12	63	111	5	3.2	1.5	0.2	6.3	6
	**All Ages** **(12 − 71 months)**	**Less than Twice a Day**	50	8	35	66	174	2.7	0.5	1.7	3.7	307
		**Two to Three Times a Day**	56	5	46	66	318	2.8	0.3	2.2	3.4	518
		**Four to Five Times a Day**	49	9	31	68	43	2.4	0.3	1.8	3.1	73
		**More than Five Times a Day**	26	12	1	51	5	1.0	0.6	**−**0.3	2.2	11
**Fruits**	**Toddler** **(12 − 47 months)**	**Never**	18	6	7	30	16	0.5	0.1	0.2	0.8	66
		**Once a Day**	39	7	26	53	78	1.3	0.2	0.9	1.7	197
		**Twice a Day**	37	6	24	50	67	1.3	0.3	0.8	1.9	159
		**Three Times a Day or More**	36	7	22	51	18	0.7	0.2	0.3	1.0	46
	**Preschooler** **(48 − 71 months)**	**Never**	100	0	100	100	13	6.3	0.5	5.2	7.3	13
		**Once a Day**	81	5	71	92	156	5.4	0.4	4.5	6.3	188
		**Twice a Day**	82	3	76	87	125	5.5	0.4	4.6	6.3	161
		**Three Times a Day or More**	87	4	78	96	67	4.6	0.6	3.4	5.7	79
	**All Ages** **(12 − 71 months)**	**Never**	25	7	11	38	29	0.9	0.2	0.5	1.3	79
		**Once a Day**	54	5	43	65	234	2.8	0.3	2.1	3.5	385
		**Twice a Day**	57	6	44	69	192	3.2	0.4	2.3	4.1	320
		**Three Times a Day or More**	61	7	48	75	85	2.6	0.4	1.9	3.3	125
**Vegetables**	**Toddler** **(12 − 47 months)**	**Never**	31	8	16	46	54	1.0	0.2	0.6	1.5	160
		**Once a Day**	33	5	23	43	97	1.2	0.2	0.8	1.7	253
		**Twice a Day**	48	9	29	66	22	1.2	0.3	0.7	1.8	44
		**More than Twice a Day**	64	14	36	91	6	0.9	0.2	0.6	1.3	11
	**Preschooler** **(48 − 71 months)**	**Never**	84	6	73	96	71	5.5	0.6	4.2	6.8	87
		**Once a Day**	84	2	79	89	230	5.4	0.3	4.8	6.0	275
		**Twice a Day**	79	7	64	94	50	4.4	0.6	3.2	5.6	65
		**More than Twice a Day**	76	14	47	105	10	4.3	1.3	1.8	6.9	14
	**All Ages** **(12 − 71 months)**	**Never**	46	8	29	62	125	2.2	0.4	1.4	3.1	247
		**Once a Day**	55	5	45	64	327	3.0	0.3	2.4	3.6	528
		**Twice a Day**	61	9	44	79	72	2.6	0.5	1.7	3.6	109
		**More than Twice a Day**	69	11	47	91	16	2.4	0.6	1.1	3.7	25
**Fast Foods**	**Toddler** **(12 − 47 months)**	**Once a Month or Less**	33	6	22	44	132	1.1	0.2	0.7	1.5	353
		**Several Times a Month**	42	5	32	52	47	1.3	0.2	0.9	1.8	111
		**Two to Three Times a Week**	0	0	0	0	0	0.0	0.0	0.0	0.0	4
	**Preschooler** **(48 − 71 months)**	**Once a Month or Less**	82	5	73	91	228	5.3	0.4	4.4	6.2	283
		**Several Times a Month**	84	3	78	90	124	5.4	0.4	4.6	6.1	149
		**Two to Three Times a Week**	100	0	100	100	9	4.7	0.4	3.9	5.5	9
	**All Ages** **(12 − 71 months)**	**Once a Month or Less**	50	6	38	62	360	2.5	0.4	1.8	3.3	636
		**Several Times a Month**	62	5	53	71	171	3.2	0.3	2.6	3.8	260
		**Two to Three Times a Week**	77	14	48	106	9	3.6	0.6	2.4	4.9	13

SE: Standard error, CI: confidence interval.

*Weighting was used to adjust the relative contribution of the respondents using known populational characteristics including age, gender, and location of residence according to Iran’s latest population census in 2016.

### Multivariate analyses of dietary patterns as determinants of ECC

Considering the methodological framework for studies using multivariate regression models to examine the relationship between multiple potential risk factors (independent variables) and a dependent outcome variable (ECC), it is essential to include background factors, such as demographic variables (age, gender, and location of residence), in addition to dental plaque, to eliminate confounding factors. This study adhered to this approach, ensuring that, despite the known associations between ECC and factors such as increasing child age, dental plaque, and living in less privileged areas, these variables were included in multivariate regression models.

Given the different characteristics and needs of the two major age groups in the study—toddlers (12–47 months) and preschoolers (48–71 months)—in terms of feeding practices and dietary habits, both bivariate and multivariate analyses were conducted and reported separately.

Table 6 reveals the factor association analysis of ECC occurrence among Iranian 1- to 5-year-olds, using bivariate logistic regression modeling (unadjusted for confounders). Variables with p-values less than 0.2 were retained for multivariate multistage logistic regression modeling (adjusted for confounders) to identify factors associated with ECC occurrence (p < 0.05) (Table 8).

**Table 6:** Factor association analysis of ECC occurrence in Iranian 1- to 5-year-olds (n = 909) using bivariate logistic regression modeling (unadjusted for confounders).

		Toddlers (12 − 47 months)					Preschoolers (48 − 71 months)				
Independent Variable	Value	OR*	SE*	p-value*	95% CI*		OR*	SE*	p-value*	95% CI*	
					Lower	Upper				Lower	Upper
**Location**	**Urban**	1.0	---	---	---	---	1.0	---	---	---	---
	**Rural**	1.4	0.6	0.344	0.7	3.1	5.2	2.4	0.001	2.1	13.0
**Gender**	**Female**	1.0	---	---	---	---	1.0	---	---	---	---
	**Male**	1.0	0.2	0.837	0.7	1.7	1.2	0.4	0.538	0.6	2.4
**Age Group**	**12 − 23 months**	1.0	---	---	---	---					
	**24 − 35 months**	6.2	2.5	<0.001	2.7	14.0					
	**36 − 47 months**	8.9	2.9	<0.001	4.6	17.3					
	**48 − 59 months**						1.0	---	---	---	---
	**60 − 71 months**						2.2	0.6	0.007	1.2	3.9
**Visible Dental Plaque**	**No**	1.0	---	---	---	---	1.0	---	---	---	---
	**Yes**	3.8	0.8	<0.001	2.5	5.7	5.7	1.6	<0.001	3.3	10.0
**Bottle Feeding Duration**	**Never**	1.0	---	---	---	---	1.0	---	---	---	---
	**1 − 12 month(s)**	0.6	0.2	0.216	0.3	1.4	0.4	0.2	0.105	0.1	1.2
	**>12 months**	1.2	0.4	0.666	0.6	2.5	0.9	0.4	0.725	0.4	2.0
**Breastfeeding Duration**	**Never**	1.0	---	---	---	---	1.0	---	---	---	---
	**1 − 18 month(s)**	1.8	0.9	0.249	0.7	4.7	1.0	0.4	0.980	0.4	2.3
	**>18 months**	3.2	1.7	0.031	1.1	9.3	2.5	1.3	0.074	0.9	6.9
**Sugar Intake Between Meals**	**Never**	1.0	---	---	---	---	1.0	---	---	---	---
	**Once a Day or Less**	2.1	0.7	0.044	1.0	4.3	0.8	0.5	0.751	0.3	2.6
	**Twice a Day**	7.0	3.3	<0.001	2.7	18.1	0.8	0.6	0.778	0.2	4.1
	**Three Times a Day or More**	9.1	6.3	0.002	2.3	36.3	1.0	1.2	0.975	0.1	10.1
**Grain Products**	**Less than Twice a Day**	1.0	---	---	---	---	1.0	---	---	---	---
	**Two to Three Times a Day**	1.3	0.3	0.355	0.8	2.0	0.8	0.3	0.550	0.5	1.5
	**Four to Five Times a Day**	0.6	0.3	0.197	0.2	1.9	0.9	0.6	0.898	0.2	3.6
	**More than Five Times a Day**	1.0	(empty)				1.3	1.5	0.845	0.1	14.4
**Fruits**	**Never**	1.0	---	---	---	---	1.0	(empty)			
	**Once a Day**	2.9	1.3	0.025	1.2	7.2	0.7	0.3	0.342	0.3	1.5
	**Twice a Day**	2.6	1.2	0.044	1.0	6.6	0.7	0.2	0.291	0.3	1.4
	**Three Times a Day or More**	2.5	0.9	0.009	1.3	5.0	1.0	(omitted)			
**Vegetables**	**Never**	1.0	---	---	---	---	1.0	---	---	---	---
	**Once a Day**	1.1	0.3	0.735	0.6	2.0	0.9	0.3	0.865	0.5	1.9
	**Twice a Day**	2.0	0.9	0.107	0.9	4.7	0.7	0.4	0.471	0.2	2.0
	**More than Twice a Day**	3.9	2.7	0.055	1.0	15.7	0.6	0.5	0.527	0.1	3.1
**Foods Rich in Protein**	**Never**	1.0	---	---	---	---	1.0	(empty)			
	**Several Times a Week**	4.1	2.1	0.008	1.5	11.4	0.4	0.3	0.272	0.1	2.2
	**Once a Day**	2.2	1.2	0.161	0.7	6.7	0.2	0.2	0.107	0.0	1.4
	**Twice a Day**	2.2	1.3	0.174	0.7	7.1	0.4	0.3	0.291	0.1	2.2
	**More than Twice a Day**	5.9	3.1	0.002	2.0	16.9	1.0	(omitted)			
**Fast Foods**	**Once a Month or Less**	1.0	---	---	---	---	1.0	---	---	---	---
	**Several Times a Month**	1.5	0.4	0.117	0.9	2.4	1.2	0.4	0.690	0.6	2.4
	**Two to Three Times a Week**	1.0	(empty)				1.0	(empty)			
**Dairy Products**	**Once a Day or Less**	1.0	---	---	---	---	1.0	---	---	---	---
	**Twice a Day**	1.0	0.2	0.933	0.6	1.6	1.0	0.4	0.935	0.4	2.4
	**Three Times a Day**	1.1	0.5	0.901	0.4	2.7	1.5	0.7	0.397	0.6	3.6
	**More than Three Times a Day**	0.5	0.4	0.421	0.1	2.9	0.4	0.3	0.223	0.1	1.7

SE: Standard Error, CI: Confidence Interval, OR: Odds Ratio.

*Weighting was used to adjust the relative contribution of the respondents using known populational characteristics including age, gender, and location of residence according to Iran’s latest population census in 2016.

**Table 7:** Factor association analysis of the dmft score in Iranian 1- to 5-year-olds (n = 909) using bivariate generalized negative binomial regression modeling (unadjusted for confounders).

		Toddlers (12 − 47 months)					Preschoolers (48 − 71 months)				
Independent Variable	Value	IRR*	SE*	p-value*	95% CI*		IRR*	SE*	p-value*	95% CI*	
					Lower	Upper				Lower	Upper
**Location**	**Urban**	1.0	---	---	---	---	1.0	---	---	---	---
	**Rural**	1.7	0.5	0.076	0.9	3.0	1.2	0.1	0.070	1.0	1.5
**Gender**	**Female**	1.0	---	---	---	---	1.0	---	---	---	---
	**Male**	1.0	0.2	0.912	0.7	1.4	1.1	0.1	0.336	0.9	1.4
**Age Group**	**12 − 23 months**	1.0	---	---	---	---					
	**24 − 35 months**	4.1	1.6	0.001	1.8	9.1					
	**36 − 47 months**	5.0	1.8	<0.001	2.4	10.3					
	**48 − 59 months**						1.0	---	---	---	---
	**60 − 71 months**						1.3	0.1	0.005	1.1	1.6
**Visible Dental Plaque**	**No**	1.0	---	---	---	---	1.0	---	---	---	---
	**Yes**	3.1	0.6	<0.001	2.0	4.7	1.9	0.3	<0.001	1.4	2.5
**Bottle Feeding Duration**	**Never**	1.0	---	---	---	---	1.0	---	---	---	---
	**1 − 12 month(s)**	0.8	0.3	0.471	0.4	1.6	0.7	0.1	0.036	0.5	1.0
	**>12 months**	1.5	0.4	0.105	0.9	2.4	1.0	0.1	0.922	0.8	1.2
**Breastfeeding Duration**	**Never**	1.0	---	---	---	---	1.0	---	---	---	---
	**1 − 18 month(s)**	1.3	0.7	0.610	0.5	3.6	1.1	0.2	0.557	0.8	1.5
	**>18 months**	2.2	1.0	0.088	0.9	5.7	1.3	0.2	0.111	0.9	1.9
**Sugar Intake Between Meals**	**Never**	1.0	---	---	---	---	1.0	---	---	---	---
	**Once a Day or Less**	1.9	0.7	0.082	0.9	3.8	0.8	0.1	0.250	0.6	1.2
	**Twice a Day**	4.4	1.7	<0.001	2.1	9.4	0.9	0.2	0.604	0.5	1.5
	**Three Times a Day or More**	3.6	1.5	0.003	1.6	8.1	0.9	0.2	0.771	0.5	1.6
**Grain Products**	**Less than Twice a Day**	1.0	---	---	---	---	1.0	---	---	---	---
	**Two to Three Times a Day**	1.4	0.3	0.118	0.9	2.2	0.8	0.1	0.023	0.7	1.0
	**Four to Five Times a Day**	0.8	0.3	0.525	0.4	1.6	0.8	0.1	0.103	0.5	1.1
	**More than Five Times a Day**	0.0	.	.	.	.	0.5	0.3	0.188	0.2	1.4
**Fruits**	**Never**	1.0	---	---	---	---	1.0	---	---	---	---
	**Once a Day**	2.7	0.9	0.005	1.4	5.3	0.9	0.1	0.206	0.7	1.1
	**Twice a Day**	2.7	0.9	0.005	1.4	5.4	0.9	0.1	0.221	0.7	1.1
	**Three Times a Day or More**	1.3	0.5	0.432	0.6	2.8	0.7	0.1	0.027	0.6	1.0
**Vegetables**	**Never**	1.0	---	---	---	---	1.0	---	---	---	---
	**Once a Day**	1.2	0.2	0.395	0.8	1.8	1.0	0.1	0.937	0.8	1.3
	**Twice a Day**	1.2	0.3	0.487	0.7	2.2	0.8	0.1	0.192	0.6	1.1
	**More than Twice a Day**	0.9	0.3	0.747	0.5	1.6	0.8	0.2	0.369	0.5	1.3
**Foods Rich in Protein**	**Never**	1.0	---	---	---	---	1.0	---	---	---	---
	**Several Times a Week**	4.4	1.9	0.001	1.8	10.7	1.0	0.1	0.964	0.8	1.2
	**Once a Day**	2.8	1.4	0.035	1.1	7.5	0.9	0.1	0.555	0.7	1.2
	**Twice a Day**	2.0	0.8	0.089	0.9	4.7	0.9	0.1	0.287	0.7	1.1
	**More than Twice a Day**	2.1	0.9	0.082	0.9	5.0	0.6	0.1	0.017	0.4	0.9
**Fast Foods**	**Once a Month or Less**	1.0	---	---	---	---	1.0	---	---	---	---
	**Several Times a Month**	1.2	0.3	0.326	0.8	1.9	1.0	0.1	0.864	0.8	1.3
	**Two to Three Times a Week**	<0.1	<0.1	<0.001	<0.1	<0.1	0.9	0.1	0.357	0.7	1.1
**Dairy Products**	**Once a Day or Less**	1.0	---	---	---	---	1.0	---	---	---	---
	**Twice a Day**	1.1	0.2	0.586	0.7	1.7	1.0	0.2	0.994	0.7	1.4
	**Three Times a Day**	1.1	0.3	0.704	0.6	2.0	1.0	0.2	0.976	0.6	1.5
	**More than Three Times a Day**	1.0	0.6	0.992	0.3	3.2	0.7	0.2	0.309	0.4	1.4

SE: Standard error, CI: confidence interval, IRR: incidence rate ratio.

*Weighting was used to adjust the relative contribution of the respondents using known populational characteristics, including age, gender, and location of residence, according to Iran’s latest population census in 2016.

**Table 8:** Factors associated with ECC occurrence in Iranian 1- to 5-year-olds (n = 909) according to multivariate multistage logistic regression modeling.

	Independent Variable	Value	OR*	SE*	p-value*	95% CI*	
						Lower	Upper
**Toddlers (12–47 months)**	**Age Group**	**12 − 23 months**	1.0	---	---	---	---
		**24 − 35 months**	4.1	1.8	0.003	1.7	10.0
		**36 − 47 months**	7.0	1.9	<0.001	4.0	12.1
	**Visible Dental Plaque**	**No**	1.0	---	---	---	---
		**Yes**	3.3	0.8	<0.001	1.9	5.5
	**Sugar Intake Between Meals**	**Never**	1.0	---	---	---	---
		**Once a Day or Less**	1.1	0.4	0.687	0.6	2.2
		**Twice a Day**	4.1	1.7	0.002	1.8	9.7
		**Three Times a Day or More**	5.8	5.0	0.046	1.0	32.5
	**Grain Products**	**Less than Twice a Day**	1.0	---	---	---	---
		**Two to Three Times a Day**	0.8	0.3	0.517	0.4	1.6
		**Four to Five Times a Day**	0.2	0.2	0.047	0.0	1.0
		**More than Five Times a Day**	1.0	(empty)			
**Preschoolers (48–71 months)**	**Location**	**Urban**	1.0	---	---	---	---
		**Rural**	4.0	1.8	0.003	1.6	9.7
	**Age Group**	**48 − 59 months**	1.0	---	---	---	---
		**60 − 71 months**	2.6	0.9	0.009	1.3	5.1
	**Visible Dental Plaque**	**No**	1.0	---	---	---	---
		**Yes**	5.0	1.2	<0.001	3.1	8.2

SE: Standard error, CI: confidence interval, OR: odds ratio.

*Weighting was used to adjust the relative contribution of the respondents using known populational characteristics, including age, gender, and location of residence, according to Iran’s latest population census in 2016.

Table 7 shows the results of the factor association analysis with the mean dmft score among Iranian 1- to 5-year-olds using bivariate generalized negative binomial regression modeling (unadjusted for confounders). Variables with p-values less than 0.2 were included in multivariate multistage generalized negative binomial regression modeling (adjusted for confounders) to identify factors associated with the mean dmft score (p < 0.05) (Table 9).

**Table 9:** Factors associated with the dmft score in Iranian 1- to 5-year-olds (n = 909) according to multivariate multistage generalized negative binomial regression modeling.

	Independent Variable	Value	IRR*	SE*	p-value*	95% CI*	
						Lower	Upper
**Toddlers (12–47 months)**	**Age Group**	**12 − 23 months**	1.0	---	---	---	---
		**24 − 35 months**	2.7	1.1	0.026	1.1	6.3
		**36 − 47 months**	3.4	1.3	0.002	1.6	7.3
	**Visible Dental Plaque**	**No**	1.0	---	---	---	---
		**Yes**	2.5	0.5	<0.001	1.7	3.6
	**Sugar Intake Between Meals**	**Never**	1.0	---	---	---	---
		**Once a Day or Less**	1.2	0.3	0.448	0.7	2.0
		**Twice a Day**	3.1	0.8	<0.001	1.8	5.1
		**Three Times a Day or More**	3.1	1.3	0.010	1.3	7.1
	**Fruits**	**Never**	1.0	---	---	---	---
		**Once a Day**	1.5	0.4	0.151	0.9	2.6
		**Twice a Day**	2.0	0.5	0.015	1.2	3.4
		**Three Times a Day or More**	0.7	0.2	0.292	0.4	1.4
**Preschoolers (48–71 months)**	**Age Group**	**48 − 59 months**	1.0	---	---	---	---
		**60 − 71 months**	1.4	0.1	0.005	1.1	1.7
	**Visible Dental Plaque**	**No**	1.0	---	---	---	---
		**Yes**	1.9	0.3	<0.001	1.4	2.5

SE: Standard Error, CI: confidence interval, IRR: incidence rate ratio.

*Weighting was used to adjust the relative contribution of the respondents using known populational characteristics, including age, gender, and location of residence, according to Iran’s latest population census in 2016.

Table 8 presents the final results of the association analysis for ECC occurrence, which was performed using multivariate multistage logistic regression modeling, separately for each major age group in the study (toddlers and preschoolers).

Among toddlers, the prevalence of ECC increases significantly with age. Specifically, children in the 36–47-month age group were 7 times more likely to have ECC than those in the 12–23-month age group (p < 0.001, OR = 7.0). Similarly, the prevalence of ECC increased with age among preschoolers (p = 0.009, OR = 2.6). The presence of visible dental plaque was significantly associated with ECC (p < 0.001), increasing the likelihood of ECC by 3.3 times in toddlers (OR = 3.3) and 5 times in preschoolers (OR = 5.0). In toddlers, the frequency of sugar intake between meals, especially twice a day (p = 0.002) or more (p = 0.046), was significantly related to ECC. Toddlers who consumed sugar twice a day between meals had a four-fold greater chance of developing ECC (OR = 4.1) compared with those who did not consume sugar between meals.

Conversely, consuming four to five servings of grains per day was associated with a five-fold reduction in ECC occurrence among toddlers (p = 0.047, OR = 0.2) compared with those who did not consume grains. Compared with living in urban areas, living in rural areas was significantly associated with ECC development only in preschoolers (p = 0.003, OR = 4.0).

Table 9 presents the final results of the association analysis with the mean dmft score, using multivariate multistage generalized negative binomial regression modeling for each major age group (toddlers and preschoolers).

Among toddlers, the dmft score increased with age. Specifically, the dmft score in the 36–47-month age group was expected to be 3.4 times higher than that in the 12–23-month age group (p = 0.002, IRR = 3.4). Similarly, in preschoolers, the dmft score also increased with age (p = 0.005, IRR = 1.4). The presence of visible dental plaque was significantly associated with higher dmft scores (p < 0.001), increasing the likelihood of a higher dmft score by up to 2.5 times (IRR = 2.5) in toddlers and 1.9 times (IRR = 1.9) in preschoolers.

In toddlers, the frequency of sugar intake between meals, especially twice a day (p < 0.001) or more (p = 0.01), was strongly associated with higher dmft scores. A toddler who consumed sugar twice a day between meals had an expected dmft score up to three times greater (IRR = 3.1) than a toddler with no sugar consumption between meals.

Additionally, consuming two servings of fruits per day was associated with a doubling of the dmft score in toddlers (p = 0.015, IRR = 2.0) compared with those who did not consume fruit.

Table 10 visualizes key findings from the bivariate and multivariate association analyses.

**Table 10:** Visual demonstration of key findings from bivariate and multivariate association analyses.

	ECC		dmft score	
	Toddlers (12 − 47) months	Preschoolers (48 − 71 months)	Toddlers (12 − 47) months	Preschoolers (48 − 71 months)
Location		**○**▲	**○**	**○**
Gender				
Age Group	**○**▲	**○**▲	**○**▲	**○**▲
Visible Dental Plaque	**○**▲	**○**▲	**○**▲	**○**▲
Bottle Feeding Duration		**○**	**○**	**○**
Breastfeeding Duration	**○**	**○**	**○**	**○**
Sugar Intake Between Meals	**○**▲		**○**▲	
Grain Products	**○**▲		**○**	**○**
Fruits	**○**		**○**▲	**○**
Vegetables	**○**			**○**
Foods Rich in Protein	**○**	**○**	**○**	**○**
Fast Foods	**○**		**○**	
Dairy Products				

**○** The p-value was obtained lower than 0.2 during bivariate regression analysis.

▲ The p-value was obtained lower than 0.05 during multivariate regression analysis.

## Discussion

This study provides an important update on the dietary determinants of ECC among young children across the age range of toddlers (12–47 months) and preschoolers (48–71 months) according to the study objectives and demographic characteristics in terms of ECC prevalence and severity.

The results of several publications on dietary risk factors for ECC are unclear. In the present study, multivariate multistage analysis was used to control for confounding factors, eliminating potential confounders and effect modifiers. This approach allowed for the retention of only those factors related to the occurrence of ECC and the dmft score, which serves as an indicator of ECC severity. Our findings revealed imperative dietary implications for oral health in early childhood as well as the importance of oral hygiene and its shadow on dietary risk factors, emphasizing the multivariable nature of dental caries.

In the present study, more than half of the children had ECC, with a prevalence of 35% among toddlers and 83% among preschoolers [26]. These rates are higher than those reported in previous studies, such as a 2005 study by Mohebbi et al., which reported that the ECC prevalence among 1- to 3-year-olds in Tehran, the capital of Iran, was between 3% and 33% [36]. According to a report by the International Association of Paediatric Dentistry (IAPD), which summarized the results of 72 studies published between 1998 and 2018, the ECC prevalence rates were 17%, 36%, 43%, 55%, and 63% in children aged 1, 2, 3, 4, and 5 years, respectively [8]. Another study by Tantawi et al., including 190 publications from 88 countries, reported that the mean ECC prevalence rates were 23.8% (SD = 14.8%) and 57.3% (SD = 22.4%) in children younger than 36 months and children aged 36 to 71 months, respectively [37]. Among children younger than 36 months, the mean prevalence of ECC was highest in North America (31.7%) and South Asia (30%) and lowest in sub-Saharan Africa (14.3%; P = 0.86). For children aged 36 to 71 months, the mean prevalence of ECC was highest in East Asia/Pacific (68.7%) and Middle East/North Africa (66.2%) and lowest in Europe/Central Asia (43.9%; P = 0.004) [37]. With a combined global ECC prevalence of 48%, the ECC prevalence varies both between and within countries. Ranging from 16% (Singapore) to 89% (China), the prevalence by continent was 30% in Africa, 48% in the Americas, 52% in Asia, 43% in Europe, and 82% in Oceania, which indicated that the distribution of ECC is not homogeneous [38]. Our results, which revealed a relatively high prevalence of ECC, highlight the urgent need for interventions.

The variation in the distribution of ECC prevalence and its severity across nations and regions could be explained by several factors, such as macroeconomic and socioeconomic conditions, genetic factors, ethnic minority populations, the availability of fluoride in drinking water or toothpaste, interventions for caries prevention, universal health coverage, the growth of gross national income, and high expenditures on health care [39].

Analysis of data from Iranian children aged 1–5 years revealed a significant increase in ECC prevalence and dmft scores with age [26]. In our study, increasing age was a definite and inseparable determinant of ECC development and higher dmft scores among both toddlers and preschoolers. However, the observed increase in caries with age is unsurprising, as prolonged exposure of teeth to a cariogenic environment naturally leads to a higher incidence of caries. This finding aligns with the study by Maklennan et al., which also highlighted that the ECC prevalence tends to increase with age, with significant findings applicable to various regions, including Iran [40].

A panel of experts has developed evidence-based best practices and recommendations for promoting healthy nutrition, eating behaviors, and feeding patterns among children aged 2–8 years. It emphasizes the importance of establishing healthy eating habits and behaviors early in life and creating a supportive food environment to foster healthy choices and prevent overeating, including fruit consumption guidelines. The recommendations also address common gaps between recommended and actual dietary intakes, promoting strategies to increase food acceptance and encourage healthy dietary patterns, such as increasing vegetable consumption and protein-rich foods, while highlighting the need for guidance on both what and how to feed children to develop healthy eating behaviors [41].

The UNICEF’s guidelines for early childhood nutrition aim to prevent malnutrition in infants and young children. They also discuss the global trend of increased consumption of sugary drinks and high-salt, high-sugar, high-fat packaged snacks among young children. This resource highlights the critical role of protein-rich foods in supporting growth and development during the complementary feeding period and emphasizes the importance of nutrient-dense diets for young children, including the introduction of diverse foods such as eggs, dairy, fish, and meat starting at six months of age [42].

The WHO guidelines on complementary feeding for infants and young children aged 6–23 months emphasize continued breastfeeding alongside the introduction of diverse solid foods starting at six months. It recommends including grains and avoiding foods with added sugars. The WHO highlights the importance of gradually increasing food consistency and variety to support healthy growth and development [43].

During our study, 39% and 37% of the participating children were revealed to consume fruits “Once a Day” and “Twice a Day”, respectively. ECC was notably more prevalent among those with higher daily fruit servings. While unadjusted bivariate analysis revealed that fruit consumption was non-inversely associated with ECC occurrence and higher dmft scores only among toddlers with “Once a Day” and “Twice a Day” fruit consumption, the multivariate multistage analysis revealed a significant association with higher dmft scores only in toddlers who consumed fruits “Twice a Day”.

A recent systematic review by Maneschy et al. (2024) on eating behaviors and dietary intake in children and adolescents examined the associations between eating behaviors and dietary intake using tools such as the Children Eating Behaviour Questionnaire (CEBQ). The review highlighted the importance of promoting healthy eating behaviors, including increased consumption of fruits and vegetables, and underscored the impact of food approach behaviors on the intake of foods rich in sugar and fats [44].

The WHO surveillance report, “How healthy are children’s eating habits?” presents insights into the dietary habits of children across Europe, including the consumption of sweets, snacks, and soft drinks and the frequency of vegetable and fruit consumption, which can help in understanding the broader context of dietary practices. The report highlights that 42.5% of children consume fresh fruit daily, which is a key indicator of healthy dietary habits. This study also provides valuable insights into current eating patterns and potential areas for dietary improvement [45].

Doubling the ECC intensity in toddlers by consuming two servings of fruit per day can be due to the higher levels of sugar content in certain fruits (such as mangoes, grapes, cherries, lychees, pineapples, bananas, figs, and palm dates) [46] or the acidic pH of others (like lemons, limes, oranges, grapefruits, pineapples, oranges, tamarinds, kiwis, and passion fruits). Certain fruits, particularly citrus varieties, contain organic acids such as citric acid. These acids can lower the pH in the oral cavity, potentially leading to enamel erosion if consumed excessively or without proper oral hygiene practices [47,48]. The acidic environment created by these fruits can accelerate the demineralization of tooth enamel, especially when combined with high sugar intake from other dietary sources [47,48].

The Centers for Disease Control and Prevention (CDC) also offers practical strategies and comprehensive information on feeding healthy foods and drinks to infants and toddlers from birth to 24 months. The CDC has also published data on fast food intake among children and adolescents in the United States, which highlights the association between fast food consumption and higher caloric intake along with poorer diet quality. They recommend that children aged 12–23 months consume 1⅔ to 2 cups of dairy daily, whereas those aged 2–4 years should consume 2 to 2 ½ cups per day [49–51].

Our study revealed an association between breastfeeding and ECC occurrence among toddlers who were breastfed for more than 18 months, using unadjusted bivariate logistic regression analysis. However, this association was no longer statistically significant during multivariate multistage logistic regression modeling when adjusted for other initially associated variables. Research findings on breastfeeding and ECC risk remain inconsistent. A systematic review by Valaitis et al. (2000) investigated the relationship between breastfeeding and ECC, providing insights into how different breastfeeding practices impact ECC risk. In light of the contradictory nature of the research findings and the weak methodology found in this systematic review, the evidence remains equivocal and does not suggest a consistent and strong association between breastfeeding and the development of ECC [52]. Similarly, a meta-analysis by Cui et al. (2017) indicated that breastfeeding for more than 12 months is associated with a greater risk of ECC [53], whereas Shrestha et al. (2024) reported a greater risk of ECC with breastfeeding beyond 12 months and nocturnal breastfeeding [54]. Avila et al. (2015) noted that while breastfeeding generally protects against ECC, prolonged breastfeeding could increase the risk for ECC [13]. These findings underscore the need for further research to clarify the role of breastfeeding in ECC development.

Our study revealed an association between bottle feeding and higher dmft scores only among preschoolers who were bottle-fed for 1–12 months, utilizing unadjusted bivariate negative binomial regression analysis. However, this association was not statistically significant according to multivariate multistage negative binomial regression modeling. Tinanoff et al. (2019) reviewed feeding practices associated with ECC, including prolonged bottle feeding and breastfeeding (beyond 12 months) and frequent and/or nocturnal feeding [8]. Similarly, the AAPD emphasizes that inappropriate bottle feeding can contribute to ECC [4]. Anil et al. presented a classification based on the severity and etiology of ECC. They concluded that inappropriate use of bottle feeding, at-will breastfeeding, or a combination of both, with or without poor oral hygiene, are significant contributors to type II ECC (moderate to severe ECC involvement) [55].

In the present study, the frequency of sugar intake between meals, especially twice a day or even more, was strongly related to both the ECC and dmft scores in toddlers. Toddlers who consumed sugar twice a day between meals presented a four-fold increase in the likelihood of ECC and a three-fold increase in dmft scores compared with those without sugar intake between meals. However an ecological study by Folayan et al. (2020) suggestes that in high-income countries (HICs), despite high per capita sugar consumption, the prevalence of ECC is relatively low. Conversely, low-income countries (LICs) exhibit high rates of ECC even with lower sugar consumption, indicating that factors other than sugar intake may play a significant role in the development of ECC [56]. The authors emphasize that the frequency of sugar consumption might be more critical than the total quantity consumed, suggesting a complex interplay between dietary habits and dental health [56].

High sugar consumption can lead to overweight and obesity, whereas a high frequency of sugar consumption is a risk factor for dental caries. This association is supported by Stephan’s curve, which shows that a decrease in salivary pH below 5.5 (critical level) after sugar consumption will cause enamel demineralization. The consumption of fermentable carbohydrates or sugars reduces the salivary pH beyond the critical pH value [57]. Yu et al. (2024) also discussed the role of high sugar consumption in the development of ECC as a significant risk factor related to the microbial etiology of ECC, emphasizing the well-established important role of dietary habits in the etiopathogenesis of ECC [58].

Additionally, socioeconomic factors are identified as determinants of behavioral contributors to the early introduction of sugar and high frequency of sugar consumption, linking lower parental education to earlier sugar exposure and, consequently, to a heightened ECC risk [59].

A strong correlation was found between the frequency of consuming foods containing free sugars (whether added or innate) twice a day or more and the occurrence and increased severity of ECC among toddlers, which calls for parents to insistently limit their child’s sugar intake.

The UNICEF’s guidance on improving young children’s diets during the complementary feeding period offers the inclusion of grain products and protein-rich foods [60]. In our study, grain consumption appeared to be protective against ECC in young children. Initial unadjusted bivariate association analyses revealed that toddlers consuming grain products “Four to Five Times a Day” had a lower ECC prevalence, whereas preschoolers consuming grains “Two to Three Times a Day” had lower dmft scores. However, after controlling for confounding factors in multivariate multistage regression modeling, only toddlers who consumed grains “Four to Five Times a Day” presented a reduced ECC risk. This means that their likelihood of developing ECC was just 20% of those with lower grain intake (less than twice a day). A cross-sectional study by Wang et al. (2022) reported contrasting results among 2- to 5-year-old Chinese children, where higher grain intake was significantly correlated with increased odds of ECC and severe ECC due to dietary imbalance and low food diversity. This study revealed that increased grain consumption was correlated with increased odds of ECC and severe ECC, whereas increased food diversity and vegetable intake were protective factors [48].

Simple carbohydrates, including foods high in refined grains (e.g., white bread, white rice, pastries, white pasta, bagels, crackers, breakfast cereals, and grain starch), are more readily and rapidly fermented by oral cariogenic microorganisms [48] than complex carbohydrates, which include whole grain-rich foods (e.g., whole grain bread, brown rice, oatmeal, quinoa, barley, whole grain pasta, popcorn, and whole grain cereals) [61]. Although our study did not differentiate between refined and whole grains, the reduced risk of ECC by one-fifth associated with the consumption of 4 to 5 servings of grain products per day likely reflects the predominant presence of whole grains in the diets of children who consume relatively higher quantities of grains.

Sandy et al. (2024), in a systematic review and meta-analysis, reported that nutritional practices, particularly low intake of fruits and vegetables, alongside other factors such as prolonged and nocturnal breastfeeding and high sugar consumption, were linked with increased caries risk [62]. A descriptive study by İnan-Eroğlu et al. (2017) assessed the correlation between diet quality and ECC in Turkish preschoolers. They reported that poor diet quality, characterized by high refined grain consumption and low dietary diversity, was linked to an increased risk of ECC [63].

In terms of fast food consumption, a majority (72%) of the children in our study had been fed fast foods “Once a Month or Less”. However, ECC was more prevalent among those children who were fed fast food more frequently, as 77% of the children who ate fast foods “Two to Three Times a Week” were affected by ECC. In support of these findings, the U.S. National Health and Nutrition Examination Survey (NHANES) (2015–2018) reported that more than one-third (36.3%) of children and adolescents aged 2–19 consumed fast food on a given day, linking frequent fast food intake with greater caloric intake and poorer diet quality [64].

### Study limitations and strengths

While the study provides valuable insights, the results must be interpreted in light of the study’s limitations. First, families’ dental service utilization could also affect children’s oral health, which might be explored in future studies. Second, our study did not differentiate between different fruits according to their sugar content or between refined and whole grains. Further studies comprising more details on the grain categorization are suggested.

However, this study has several strengths. The representative sample was ensured through an equal-sized, stratified, multistage random sampling approach, which enhanced the reliability of the findings. Additionally, using a reliable index and statistical analysis models appropriate for the multilevel nature of the sampling, along with a weighted process, allowed us to achieve results close to reality and generalize these findings to the broader population.

## Conclusion

This study examined possible dietary risk factors for ECC in Iranian children, such as increased sugar intake and fruit consumption, while balancing diet with grain consumption played a protective role (Table 10).

A strong correlation was found between the consumption frequency of foods containing free sugars (either added or innate) twice a day or more and the occurrence and increased severity of ECC among toddlers, which calls for parents to insistently limit their child’s sugar intake.

While fruits are an essential part of a healthy balanced diet, to reduce the risk of ECC associated with high-sugar and acidic fruits, it is recommended that parents delay the introduction of such foods into their children’s diets and encourage healthier eating habits, including promoting whole fruits over juices.

Unlike simple carbohydrates, including refined, processed, or starchy grain products, complex carbohydrates such as whole grains take longer to break down and do not pose the same immediate risk for caries. Although complex carbohydrates can still contribute to dental issues if they remain stuck on teeth, their overall impact is less severe compared to simple carbohydrates.

Despite the importance of dietary patterns, particularly in toddlers, the role of oral hygiene and the multifactorial nature of the disease should not be neglected when identifying routes for developing future targeted and effective preventive strategies.

## Supporting information

S1 Data The survey mini data file. (XLSX)
